# Periprosthetic joint infection in unicompartmental knee arthroplasty: treatment options and outcomes. What is the current evidence in literature?

**DOI:** 10.1007/s00402-022-04414-4

**Published:** 2022-03-18

**Authors:** Andrea Zanirato, Luca Cavagnaro, Francesco Chiarlone, Emanuele Quarto, Matteo Formica

**Affiliations:** 1 IRCCS Ospedale Policlinico San Martino—Clinica Ortopedica, Largo Rosanna Benzi 10, 16132 Genova, GE Italy; 2 DISC Dipartimento Di Scienze Chirurgiche e Diagnostiche Integrate, Viale Benedetto XV 6, 16132 Genova, GE Italy; 3 Joint Replacement Unit, Ortopedia e Traumatologia 2––Ospedale Santa Corona, Viale 25 Aprile, 38, 17027 Pietra Ligure, SV Italy

**Keywords:** Unicompartmental knee arthroplasty, Periprosthetic joint infection, UKA infection, 2-stage exchange, 1-stage exchange, DAIR

## Abstract

**Introduction:**

Periprosthetic joint infections (PJI) following unicompartmental knee arthroplasties (UKAs) will increase. The aim of this review is to evaluate current evidence regarding treatment options, complications, clinical and radiological outcomes of PJI management in UKAs.

**Methods:**

A systematic review of English literature was performed. Retrospective and prospective studies providing treatment options, complications, clinical and radiological outcomes of PJI following UKAs were included. PJI type, treatment, survival rate with no reoperation for infection and survival rate with no reoperation for any cause were evaluated.

**Results:**

Eleven articles were included. Three studies focusing on PJI following UKA (45 cases) report a survival rate with no reoperation for infection of 68.9% and a survival rate with no reoperation for any cause of 48.9%. Eight articles concerning UKA failure modes (28 cases) overestimate survival rate with no reoperation for infection (88.9%) and survival rate with no reoperation for any cause (88.9%) (*p* < 0.05). DAIR reports a rate of infection eradication failure ranging from 43.8 to 100%. 1SE allows for a survival rate with no reoperation for infection of 100%. 2SE reports a rate of infection eradication failure ranging from 0 to 12.5%. A high rate of early aseptic reoperation is reported, despite infection eradication (20% in DAIR; 28.5% in 2SE).

**Conclusions:**

Treatment strategy is determined by symptom timing, PJI type (acute vs chronic), causative organism, patient’s comorbidities. A longer duration of PJI or severe host and extremity status seems to require 2SE or 1SE. Patients who have a shorter duration of PJI could receive DAIR.

## Introduction

Unicompartmental knee arthroplasty (UKA) is an increasingly popular surgical procedure which, subject to proper indications, allows to significantly improve knee function and patient satisfaction, providing long-term survival rates above 90% [1, 2]. Analyzing the different national joint replacement registries, UKA usage is reported at 2–12% in clinical practice [3–6].

The number of unicompartmental knee arthroplasties (UKAs) performed is progressively increasing in light of the good clinical and radiological outcomes. Between 1998 and 2005, UKAs performed in the United States increased almost eightfold [7].

The periprosthetic joint infection (PJI) is a devastating, well-known complication, which occurs in 1–2% of cases following total knee arthroplasty (TKA) [8, 9]. According to national registries, UKA presents a lower rate of PJI: 0.1–0.8% of total cases [10]. Nevertheless, a recent systematic review reported a mean 4.2–4.8% rate of PJI usually occurring shortly after surgery (< 6 months) [11]. As observed in TKAs, registry data are often inaccurate, particularly with reference to PJI, which probably leads to underestimating septic complications after joint replacement. Large case series from tertiary referral centers suggest for a more in-depth assessment of revision causes due to standardized definitions [12].

Considering this revised incidence of PJI following UKAs and the estimated further increase in PJI cases, due to the proportional annual rise in UKAs performed, better defining the management options and the consequent clinical results of PJI in UKAs is a matter of urgency.

The pathogenesis of PJI in patients with UKAs implies the simultaneous occurrence of implant-related infection and native septic knee arthritis. This condition determines a unique clinical scenario: the simultaneous infection of prosthesis components, native cartilage-bone tissue and biomechanically fundamental soft tissues. The Society of Unicondylar Research and Continuing Educations published a multicenter study to confirm the validity of laboratory tests and the corresponding cut-off values for the diagnosis of PJI following UKA [13]. Nevertheless, guidance for managing PJI following UKA is limited, as reflected by the 2018 International Consensus Meeting (ICM) on PJI, where the debridement and implant retention (DAIR) approach was condoned in both acute and chronic conditions, based on limited evidence [14, 15].

Against this background, the aim of this paper is to systematically review the literature to evaluate current evidence regarding treatment options, complications, clinical and radiological outcomes of PJI management in UKAs.

## Materials and methods

A systematic review of English literature was performed using PRISMA as guidance to evaluate current evidence regarding treatment options and outcomes of PJI in UKA [16].

An electronic search was performed in the databases of MedLine, EMBASE, CINAHL and the Cochrane Library on 1 February 2021, by entering the following keywords and combination of keywords: “unicompartmental knee arthroplasty”, “unicondylar knee arthroplasty”, “partial knee arthroplasty”, “unicompartmental knee replacement”, “UKA”, “partial knee replacement”, “UKAs failure”, “periprosthetic joint infection” and “infection”.

We included studies providing treatment options, complications, clinical and radiological outcomes of PJI after UKA, retrospective or prospective clinical studies including randomized controlled trials and non-randomized trials, cohort studies, case-control studies and case series.

We excluded articles that did not provide clear data about the management strategy of PJI following UKA and of the related complications, clinical and radiological results, experimental, biomechanical or in vitro studies, surgical technique papers, case reports, reviews or meta-analyses and registry studies.

Once duplicates were removed, the articles were evaluated for relevance based on the title and abstract. The selected articles were then assessed using pre-defined inclusion and exclusion criteria. The references section of the identified articles was checked in order not to omit any further relevant articles. With the support of a senior author to refer to in case of uncertainties, one author conducted the selection of articles.

The studies were assessed for level of evidence (LOE) according to the Oxford Centre for Evidence-Based Medicine 2011 Levels of Evidence (OCEBM) [17].

The following data, when available, were extracted from the included articles: number of treated knees, number of patients, mean age of the population (years), time from UKA implantation to symptoms, timing between symptoms and treatment, PJI type, causative organism, patient’s comorbidities, antibiotic therapy used (in the perioperative period and chronic suppressive therapy), type of treatment applied and further treatment.

PJI type (acute post-operative, chronic, and acute hematogenous) was defined in line with the criteria established by the ICM on PJI [14, 15, 18].

Data drawn from the selected studies were firstly categorized according to PJI type considering ICM criteria. Subsequently, each PJI type was classified according to the treatment strategy that was adopted. If categorization of PJI type was not possible, the analysis was conducted based solely on the type of treatment adopted.

The survival rate with no reoperation for infection and survival rate with no operation for any cause according to Chalmers et al. [14] were calculated as primary endpoints. Treatment success was defined as no further surgical intervention for infection.

Categorical variables were expressed as number of cases or percentage. Continuous variables were reported as mean ± standard deviation (SD). Statistical analysis was conducted using ϰ^2^ analysis or Fisher’s exact test for categorical variables and independent *t* test for continuous variables. Statistical significance was established at *p* < 0.05.

## Results

**Fig.1:**
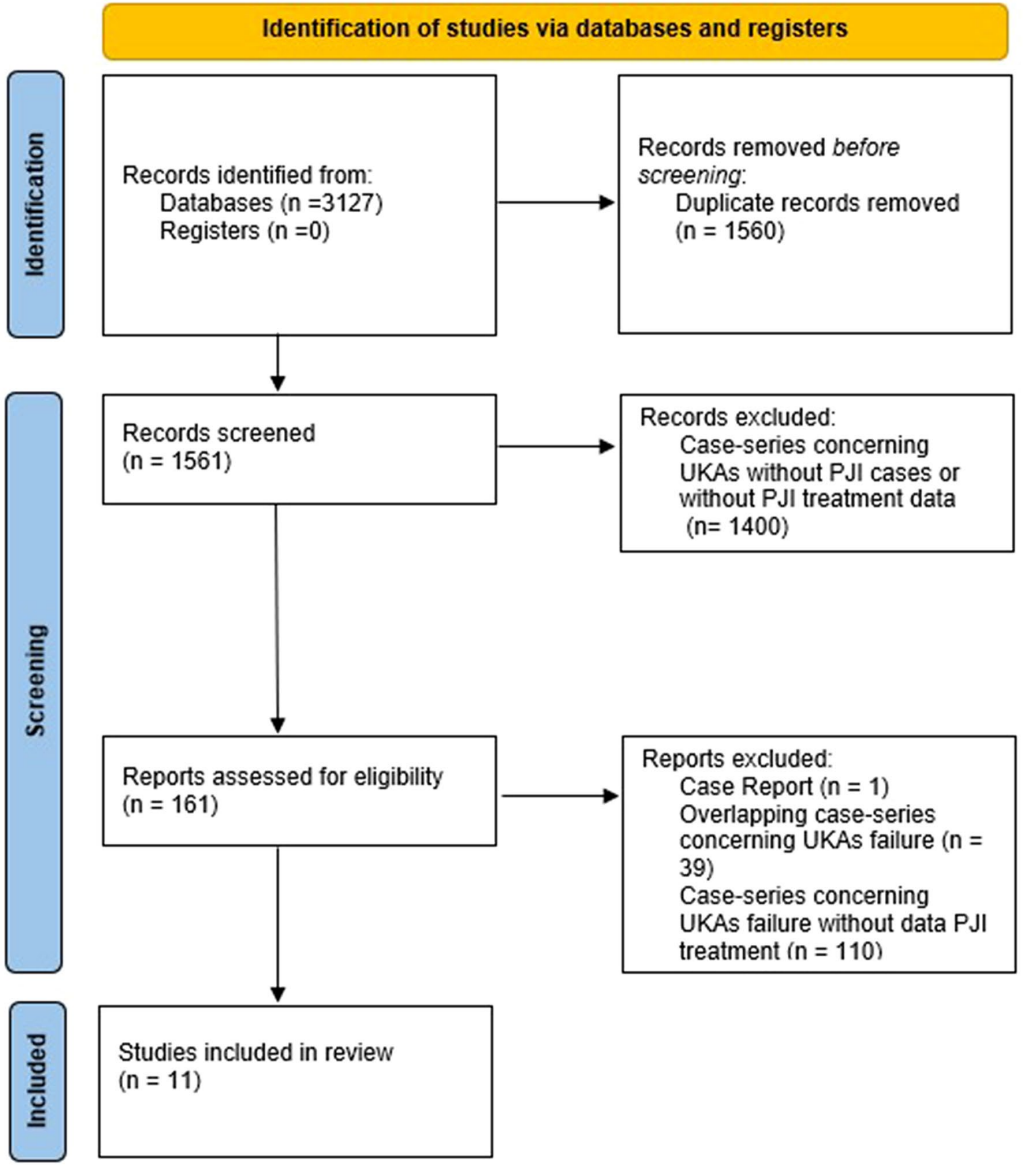
The PRISMA 2020 flow diagram illustrates the studies that have been identified, included and excluded as well as the reason for exclusion

A total of 11 articles were eventually included in the systematic review. The PRISMA 2020 diagram illustrates the studies that were identified, included and excluded, as well as the rationale for exclusion (Fig. 1).

Articles classifying PJI based on ICM criteria were included in Group A. Articles reporting only treatment strategy adopted without definition of PJI type were included in Group B. More specifically, Group A included 3 papers, LOE IV, specifically focusing on PJI management following UKAs [14, 19, 20]. Group B included 8 papers: one paper (LOE IV) reporting treatment of a mixed population of TKAs-UKAs PJI (data of UKAs PJI subpopulation were extrapolated to be included in the current analysis) [21] and 7 articles (LOE III) analyzing UKAs failure modes and describing management of PJI as possible complication [22–28]. Data drawn from the articles that were included are shown in Table 1.

**Table 1:** Articles included

	PJI UKAs (pts)	Population age (years)	Time UKA—PJI symptoms (days)	Infection type	Initial treatment	Survival rate with no reoperation for infection	Survival rate with no reoperation for any cause	Mean FU (years)
Group A								
Chalmers et al. [14]	21 (21)	66 (51–87)	199.9 ± 321.0	14 (67%) acute postop	16 (76%) DAIR	76% (2 years FU)	57% (5 years FU)	3 (1–9)
				3 (14%) acute hematog	4 (19%) 2SE			
				4 (19%) chronic	1 (5%) 1SE			
Hernandez et al. [19]	15 (15)	58 (41–82)	287.1 ± 571.5	5 (33.3%) acute postop	11 (73.3%) DAIR	71% (5 years FU)	49% (5 years FU)	4 (2–6)
				5 (33.3%) acute hematog	4 (26.7%) 2SE			
				5 (33.3%) chronic				
Labruyere et al. [20]	9 (9)	67 (36–83)	–	9 (100%) chronic	5 (55.6%) DAIR	55.6%	55.6%	5 (3–8)
					4 (44.4%) 1SE			
Group B								
Burger et al. [22]	1 (1)	–	–	–	1SE	100%	100%	–
Mohammad et al. [23]	3 (3)	–	–	–	1 (33%) Arthroscopic debridement and washout	33%	33%	–
					2 (66%) DAIR			
Pandit et al. [24]	6 (5)	–	–	–	6 (100%) 2SE	100%	100%	–
Price et al. [25]	5 (5)	–	–	–	5 (100%) 1SE	100%	100%	–
Saxler et al. [26]	1 (1)	–	–	–	1 (100%) Synoviectomy and Gentamicin Chain	100%	100%	–
Singer et al. [21]	6 (6)	–	–	–	6 (100%) 1SE	100%	100%	–
Winnock de Grave et al. [27]	4 (4)	–	–	–	4 (100%) 2SE	100%	100%	–
Xue et al. [28]	2 (2)	–	–	–	2 (100%) 2SE	100%	100%	–

*DAIR* debridement and implant retention, *FU* follow-up, *PJI* periprosthetic joint infection, *Preop* preoperative, *TKA* total knee arthroplasty, *1SE* one-stage revision procedures, *2SE* two-stage revision procedures, *(-)* not reported

In the second group (Group B), the lack of data concerning PJI type, timing between symptoms and treatment, type of infections according to ICM, patients’ comorbidities, antibiotic protocol used, standardization of diagnostic criteria according to ICM criteria and treatment options was evident.

### Group A

Forty-five cases of PJI following UKAs were reported with a mean FU of 4 ± 1 years: nineteen were classified as acute post-operative, 18 as chronic and 8 as acute hematogenous, according to the criteria established by the ICM on PJI. All authors stated they had closely worked with infectious disease specialists [14, 19, 20]. The case series of Labruyere et al. [20] is homogeneous as it includes only chronic PJI following UKAs; in 5 patients, a DAIR approach was previously performed in other orthopedic centers (for such reason, these cases were categorized as primarily treated with DAIR with infection recurrence in this review). Chalmers et al. and Hernandez et al. [14, 19] reported in detail Musculoskeletal Infection Society (MSIS) major and minor diagnostic criteria for PJI diagnosis [14, 19]. Labruyere et al. did not report the mean time between UKA implant and PJI symptoms nor McPherson host grade of patients [20]. DAIR procedures consisted in complete synovectomy and liner exchange in all cases reported by Chalmers et al. [14]; Hernandez et al. reported polyethylene exchange only in 73% of cases treated with DAIR [19]. Two-stage revision procedures (2SE) implied the removal of prosthetic components, completing femoral and tibial bone resection cuts for subsequent TKA, placement of a high-dose antibiotic spacer, 6 weeks of intravenous or high-dose oral antibiotics, and definitive TKA insertion, once persistent infection had been ruled out [14, 19]. Chalmers et al. [14] used an articulating spacer, whereas Hernandez et al. reported usage of non-articulating spacer [19]. One-stage revision procedures (1SE) consisted in complete synovectomy, removal of prosthetic components, total knee replacement cuts and placement of definitive TKA components in the same operative setting [14, 20]. Labruyere et al. described an intravenous antibiotic protocol of 6 weeks, followed by additional 6 oral weeks after 1SE revision to TKA [20].

The International Knee Society (IKS) scores or Knee Society Scores (KSS) were determined to evaluate the clinical outcome in 2 studies: Hernandez et al. [19] reported a median KSS improving from 73 (50–93) to 94 (55–100); Labruyere et al. reported a post-operative median Knee score of 75 (47–100) and a median function score of 65 (10–90) [20].

In Group A, the global survival rate with no reoperation for infection was 68.9% and the survival rate with no reoperation for any cause was 48.9%.

Analyzing treatment results based on PJI type, the global survival rate with no reoperation for infection was 63.2% in acute post-operative PJI, 87.5% in acute hematogenous PJI and 66.6% in chronic PJI. The survival rate with no operation for any cause was 42.1% in acute post-operative PJI, 50% in acute hematogenous PJI and 55.6% in chronic PJI.

A further analysis was performed to investigate statistically significant differences (ϰ^2^ analysis or Fisher’s exact test) for successful infection eradication due to McPherson Host Grade, causative organisms and MSIS diagnostic criteria. No significant difference was observed (*p* > 0.05).

With regards to the treatment strategy adopted, DAIR was the first approach adopted in 71.1% of cases in Group A. The global survival rate with no reoperation for infection with DAIR was 56.3%, whereas the survival rate with no reoperation for any cause was 37.5% and the rate of infection eradication failure was 43.8%. Evaluating 2SE approach results, the survival rate with no reoperation for infection was 87.5% and the survival rate with no reoperation for any cause was 62.5%. Analyzing outcomes of 1SE approach, the survival rate with no reoperation for infection was 100% and the survival rate with no reoperation for any cause was 100%. A high rate of early aseptic reoperations was reported: 20% in DAIR subgroup and 28.5% in 2SE subgroup. These data suggest that a strict follow-up should be observed, to identify degeneration progression of native knee compartments and TKA aseptic complications at an early stage.

A further analysis on treatment results (DAIR, 1SE and 2SE) was performed based on PJI type according ICM criteria and is shown in Table 2.

**Table 2:** Treatment results according PJI type

Infection type	Initial treatment	Survival rate with no reoperation for infection	Survival rate with no reoperation for any cause
Acute post-operative PJI (19 cases)	DAIR + chronic suppressive antibiotic therapy (11 cases; 57.9%)	63.6%	36.4%
	DAIR without chronic suppressive therapy (6 cases; 31.6%)	66.6%	50%
	2SE without chronic suppressive therapy (2 cases; 10.5%)	50%	50%
Acute hematogenous PJI (8 cases)	DAIR + chronic suppressive antibiotic therapy (5 cases; 62.5%)	100%	80%
	DAIR without chronic suppressive therapy (2 cases; 12.5%)	0%	0% (2 consecutive 2SE)
	2SE without chronic suppressive therapy (1 case; 25%)	100%	50%
Chronic PJI (18 cases)	DAIR without chronic suppressive therapy (5 cases; 27.8%)	0%	0%
	1SE without chronic suppressive therapy (4 cases; 22.2%)	100%	100%
	DAIR + chronic suppressive antibiotic therapy (4 cases; 22.2%)	75%	50%
	2SE without chronic suppressive therapy (3 cases; 16.7%)	100%	100%
	1SE without chronic suppressive therapy (1 case; 5.6%)	100%	100%
	2SE + chronic suppressive antibiotic therapy (1 case; 5.6%)	100%	0%

*DAIR* debridement and implant retention, *FU* follow-up, *PJI* periprosthetic joint infection, *Preop* preoperative, *TKA* total knee arthroplasty, *1SE* one-stage revision procedures, *2SE* two-stage revision procedures

### Group B

Twenty-eight cases of PJI following UKAs were selected. The mean implant age was 1.9 ± 2.2 years. Only in 2 case series, the causative infectious organisms were reported [24, 27]. Only two authors reported the antibiotic protocol used [21, 22]. Data about timing between symptoms and treatment, type of infections and patients’ comorbidities were lacking. In Group B, the global survival rate with no reoperation for infection was 88.9%, whereas the survival rate with no reoperation for any cause was 88.9%.

In 12 cases, a two-stage approach was successfully adopted, without subsequent aseptic revisions reported (survival rate with no reoperation for infection of 100% and with no reoperation for any cause of 100%). Likewise, in 12 cases a one-stage revision procedure was performed with a survival rate with no reoperation for infection of 100% and a survival rate with no reoperation for any cause of 100%. Saxler et al. reported a case treated with synoviectomy and positioning of gentamicin chain [26]. Mohammad et al. reported one case of arthroscopic debridement and washout with infection resolution [23]. Conversely, other 2 cases were treated with open debridement, washout and liner exchange (DAIR) with infection recurrence requiring 1SE and 2SE revision to TKA [23].

No data about clinical results were reported in this group.

Table 1 shows the data drawn from Group B studies.

### Group A vs. Group B

When comparing Group A and Group B, statistically significant differences were noted in the survival rate with no reoperation for infection (68.9% in Group A vs. 88.9% in Group B; *p = *0.0008) and the survival rate with no operation for any cause (48.9% in Group A vs. 88.9% in Group B; *p = *0.0001). Group B overestimates outcomes of PJI treatment in UKAs.

As previously reported, several biases and inaccuracies were noted in Group B. The lack of data concerning PJI type, timing between symptoms and treatment, type of infections according to ICM criteria, patients’ comorbidities, antibiotic protocol, standardization of diagnosis and treatment options, could limit and distort the analysis. Conversely, the articles included in Group A provided details of these important data with a mid- to long-term follow-up (mean FU 4 ± 1 years).

With reference to the type of treatment adopted, DAIR was the most frequently adopted approach in Group A (71.1% of cases): the global survival rate with no reoperation for infection with DAIR was 56.3%, whereas the survival rate with no operation for any cause was 37.5% (about 20% of patients required an early aseptic conversion to TKA). In Group B, DAIR was reported only in 7.1% of cases and treatment was unsuccessful in all cases. The failure rate of infection eradication was 43.8% in Group A and 100% in Group B (*p* < 0.0001).

When comparing Group A and Group B for 2SE approach, statistically significant differences were noted in the survival rate with no reoperation for infection (87.5% in Group A vs. 100% in Group B; *p = *0.0003) and the survival rate with no reoperation for any cause (62.5% in Group A vs. 100% in Group B; *p = *0.0001). In Group A, 28.5% of patients with infection eradication had to undergo a revision TKA due to aseptic loosening in short-term FU (*p = *0.0001 compared to Group B where no cases of aseptic TKA revisions were reported).

Analyzing 1SE approach, the survival rate with no reoperation for infection was equal to 100% in both Group A and Group B (*p* > 0.05). Similarly, the survival rate with no reoperation for any cause was equal to 100% in both groups (*p* > 0.05).

## Discussion

Unicompartmental knee arthroplasty is an increasingly popular surgical treatment for single-compartment knee osteoarthritis. Given the rise in PJI diagnosis following UKAs, septic complications are likely to become an increasingly common clinical problem.

Clear guidance for managing PJI following UKA does not exist and is limited to the 2018 International Consensus Meeting (ICM) on PJI, where the debridement and implant retention (DAIR) approach was condoned in both acute and chronic situations [14, 15, 18].

Several approaches were presented in literature, which are associated with chronic suppressive antibiotic therapy: DAIR, 2SE and 1SE. The goals of surgical treatment include, both at implant and native knee compartment level, decompression, lavage, debridement and, in some cases, synovectomy [14, 19, 29].

The studies reporting UKAs failure mode (Group B) are characterized by the limited possibility to describe PJI type according to ICM criteria, timing between symptoms and treatment, patients’ comorbidities, antibiotic protocol and standardization of diagnosis and treatment options. Furthermore, case series focusing on UKAs failure modes appear to overestimate results of PJI treatment in terms of survival rate with no reoperation for infection and survival rate with no reoperation for any cause compared to studies mainly focusing on the management of septic complications following UKAs. This review shows a high rate of failure in infection eradication and high percentage of subsequent revision surgeries due to prosthetic aseptic loosening. The periprosthetic joint infection following UKA appears as an insidious clinical scenario, the management of which is to be deemed as very complex and often unsatisfactory.

Technically, it is possible to note a higher degree of infected tissue excision in UKA PJI, compared to TKA PJI [20]. Nevertheless, it is important to highlight that pathogenesis of PJI in patients with UKAs requires a simultaneous occurrence of implant-related infection and septic arthritis of native knee [14, 19]. The cartilage plays a pivotal and crucial role in the management of PJI following UKAs. Similarly to native knee arthritis, the infection determines an early cartilage damage (damage starts about 8 h after infection) [14, 19, 29]. A cartilage damage can be also determined by toxicity of irrigation solution during articular lavages or DAIR procedures and debridement procedures [14].

The damage of native cartilage and instability due to cruciate ligaments impairment (caused by infection and debridement), in spite of infection eradication, could theoretically determine subsequent aseptic degenerative arthritis of other compartments and consequently UKA failure after DAIR [14, 19].

In the analysis conducted on the data drawn from Group A, in spite of infection eradication, a high rate of early aseptic reoperations was noted (20% in DAIR subgroup). These data suggest that a strict follow-up should be observed to identify degeneration progression of native knee compartments at an early stage.

Furthermore, the chondrocytes and sub-chondral osteocytes could potentially hinder infection eradication. Especially in chronic situations, internalization of bacteria (i.e. Staphylococcus aureus) by osteocytes and chondrocytes is a well-known mechanism of disease recurrence [30] and may explain the rapid recurrence of infection even with extensive antibiotic administration and accurate articular lavage in DAIR procedures.

The rate of infection eradication failure with DAIR was 43.8% in Group A and 100% in Group B.

A thorough debridement of articular cartilage may thus be relevant for infection eradication [19]. However, taking account of the evidence on native septic arthritis, cartilage debridement and arthroscopic irrigation alone have been reported to be equally successful [31].

In 1SE and 2SE approaches, implant removal and accurate surgical debridement of all knee compartments were crucial in surgery [14, 19, 20]. The use of single-compartment spacers should be avoided in 2SE. In the 2SE approach, the rate of infection eradication failure was 13.5% in Group A and 0% in Group B. In 1SE approach, the survival rate with no reoperation for infection was 100% both in Group A and Group B.

As observed in this review, a high rate of early aseptic reoperations after 2SE approach was observed despite infection eradication (28.5% in Group A). These data suggest that a strict follow-up should be observed to identify TKA aseptic complications at an early stage.

Current evidence in literature suggests that early diagnosis and treatment of PJI are relevant for the outcomes to be successful and for limiting morbidity in the native knee in case of UKA [14, 19].

The timing of symptoms, type of PJI (acute vs chronic), causative organism, patient comorbidities and local extremity grade are the main factors influencing decision regarding treatment [14, 19]. The literature suggests that patients with a longer duration of PJI or more severe host and extremity status should receive two-stage or one-stage exchange. Patients who have a shorter duration of PJI could receive DAIR [19]. Nevertheless, a difference of results can be noted based on type of acute infection. In acute hematogenous PJI, DAIR with chronic antibiotic therapy determines a survival rate with no reoperation for infection of 100%. In acute post-operative PJI, DAIR determines a survival rate with no reoperation for infection of approximately 60%.

In chronic PJI after UKA, one-step conversion to TKA can be considered if: (a) the causative organism is identified preoperatively in joint aspirate, (b) the causative organism is susceptible to antibiotics and (c) a complete excision of the infected tissues can be performed [20].

Although recent literature does not recommend DAIR in chronic TKA infections, this suggestion is not strictly adopted in UKA chronic PJI. According to this review, (failure of infection eradication was 43.8% in Group A and 100% in Group B), the authors cautiously recommend using DAIR procedure in acute UKA infections with careful preoperative screening and proper case-by-case analysis. Different preoperative scoring systems are available [32]. Even if these scores are not specifically validated for UKA infections, they could represent a useful tool to properly predict and maximize success rate of DAIR procedure.

As demonstrated by current analyses, failure of infection eradication could require multiple subsequent surgeries. In such clinical settings, a higher level of constraint and diaphyseal stems should be adopted due to extensive debridement and bone loss, mainly in cases of subsequent 2SE revision TKA due to recurrent PJI [14, 19, 20, 33]. Some of the articles included in the review reported the use of metaphyseal devices [14, 19]. In the last decade, metaphyseal cones and sleeves have been widely used in revision TKA, including PJI, to obtain implant stability and joint line reconstruction [34, 35].

To our knowledge, this is the first systematic review that analyses and compares results of different treatments for PJI following UKAs.

The review process undoubtedly has several limitations. Data analysis was difficult, due to limited literature available. Moreover, evidence concerning PJI treatment from Group B was characterized by poor quality of evaluation, high amount of biases and methodological inaccuracies. Only three articles of LOE IV directly focused on the management of UKAs PJI.

Further high-quality long-term studies could better clarify the results of different approaches to PJI in UKAs.

## Conclusion

UKA infection is a unique clinical scenario where both prosthesis and native cartilage are involved. The 2018 International Consensus Meeting (ICM) on PJI condoned DAIR approach in both acute and chronic situations. Nevertheless, DAIR procedures show a rate of infection eradication failure ranging from 43.8 to 100%. 1SE and 2SE procedures provide better results in infection eradication. Current evidence in literature suggests that early diagnosis and treatment of PJI are pivotal to successful outcomes. A longer duration of PJI or more severe host and extremity status seem to suggest 2SE or 1SE. Patients who have a shorter duration of PJI could receive DAIR.
